# Effect of Vanadium Addition on Solidification Microstructure and Mechanical Properties of Al–4Ni Alloy

**DOI:** 10.3390/ma17020332

**Published:** 2024-01-09

**Authors:** Xu Chen, Ji Chen, Weiguo Xi, Qizhou Cai, Jingfan Cheng, Wenming Jiang

**Affiliations:** State Key Laboratory of Materials Processing and Die & Mould Technology, School of Materials Science and Engineering, Huazhong University of Science and Technology, Wuhan 430074, China; d202180453@hust.edu.cn (X.C.); m202270951@hust.edu.cn (J.C.); m202170966@hust.edu.cn (W.X.); d201377244@hust.edu.cn (J.C.); wmjiang@hust.edu.cn (W.J.)

**Keywords:** Al–4Ni alloy, vanadium, thermal analysis, solidification process, microstructure evolution, mechanical properties

## Abstract

The effects of vanadium addition on the solidification microstructure and mechanical properties of Al–4Ni alloy were investigated via thermodynamic computation, thermal analysis, microstructural observations, and mechanical properties testing. The results show that the nucleation temperature of primary α-Al increased with increased vanadium addition. A transition from columnar to equiaxed growth took place when adding vanadium to Al–4Ni alloys, and the average grain size of primary α-Al was reduced from 1105 μm to 252 μm. When the vanadium addition was 0.2 wt%, the eutectic nucleation temperature increased from 636.2 °C for the Al–4Ni alloy to 640.5 °C, and the eutectic solidification time decreased from 310 s to 282 s. The average diameter of the eutectic Al_3_Ni phases in the Al–4Ni–0.2V alloy reduced to 0.14 μm from 0.26 μm for the Al–4Ni alloy. As the vanadium additions exceeded 0.2 wt%, the eutectic nucleation temperature had no obvious change and the eutectic solidification time increased. The eutectic Al_3_Ni phases began to coarsen, and the number of lamellar eutectic boundaries increased. The mechanical properties of Al–4Ni alloys gradually increased with vanadium addition (0–0.4 wt%). The Al–4Ni–0.4V alloy obtained the maximum tensile strength and elongation values, which were 136.4 MPa and 23.5%, respectively. As the vanadium addition exceeded 0.4 wt%, the strength and elongation decreased, while the hardness continued to increase. Fracture in the Al–4Ni–0.4V alloy exhibited ductile fracture, while fracture in the Al–4Ni–0.6V alloy was composed of dimples, tear edges, and cleavage planes, demonstrating mixed ductile–brittle fracture. The cleavage planes were caused by the primary Al_10_V and coarse Al_3_Ni phases at the boundary of eutectic cells.

## 1. Introduction

The applications of cast heat-resistant aluminum alloys in automobiles are important for cylinder blocks, cylinder heads, and cylinders in engines. With the development of automobiles towards high-power, high-energy utilization, and low pollution emissions, higher requirements have been put forward for the heat-resistant performance of cast aluminum alloys [1,2,3]. Conventional cast heat-resistant aluminum alloys, such as Al–Si–Mg systems [4,5] and Al–Cu–Mg systems [6,7], show sharp increases in their alloying element diffusion, and decreases in their solid solution strengthening at temperatures of 200 °C or above. The strengthening phases such as Al_2_Cu and Mg_2_Si coarsen significantly at high temperatures, resulting in a decreased pinning effect on dislocations and grain boundaries, while the aging metastable phases transform into a stable phase and no longer have a lattice-matching relationship with the matrix [8,9]. Therefore, conventional cast heat-resistant aluminum alloys have difficulty meeting the high explosive pressures and high service temperatures required for high-power engines, and it is of great significance to develop new heat-resistant aluminum alloys.

Aluminum alloys containing an Al–Al_3_Ni eutectic structure (eutectic temperature 639.9 °C) have great potential to replace conventional aluminum alloys for high-temperature applications. The Al_3_Ni eutectic phases (Pnma crystal structure, with lattice parameters, *a* = 0.66115 nm, *b* = 0.73364 nm, and *c* = 0.48118 nm [10]) are distributed with a fine rod-like morphology, which tends to repel the matrix dislocations due to their higher elastic modulus [11]. Moreover, the interfaces between Al_3_Ni and the aluminum matrix are coherent, which increase the coarsening resistance at high temperature, and make the Al_3_Ni phases have excellent chemical and thermal stability up to 500 °C [12,13]. In addition, eutectic Al–Ni alloy has good castability due to the high-volume fraction of its Al_3_Ni phase (~10 vol%), and has a low tendency to hot crack.

However, the as-cast hypoeutectic Al–Ni alloys are prone to form coarse primary α-Al dendrites and eutectic structures, and the solubility of Ni in α-Al is very low, which restricts the performance of Al–Ni alloys. The addition of transition elements, such as Cr, Mn, Co, etc., can refine the primary α-Al dendrites and modify the eutectic phases, as well as provide a suitable as-cast microstructure for improving high-temperature performance. Pandey et al. [14] studied the effect of Cr (0.5–0.7 at%) on the microstructure and properties of Al–Ni alloys. The results showed that the ternary eutectic alloys display a complex microstructure containing hierarchically arranged plate and rod-like intermetallic phases that exhibit extraordinary mechanical properties. The study by Gan et al. [15] shows that trace amounts of Co addition (0.1 at%) could refine the Al_3_Ni fibers under both normal gravitational and super-gravitational fields. Sankanit et al. [16] found that the eutectic Al_3_Ni phases could be transformed into a Al_9_(Ni,Mn)_2_ phase by adding Mn into Al–Ni alloy; the Al–4Ni–1Mn–0.36Sc–0.06Zr alloy exhibited excellent thermal stability up to a maximum temperature of 350 °C. Rare earth elements such as Yb [17], Sc [18], and Er [19] could effectively refine the microstructure and improve the properties of Al–Ni alloys.

The transition element vanadium can refine primary α-Al in aluminum alloys through constitutional supercooling or heterogeneous nucleation of vanadium-bearing compounds. Moreover, the low diffusion coefficient and solubility of vanadium in aluminum alloys can also improve their high-temperature performance [20,21]. Wang’s study [22] found that additions of 0.3 wt% V and 0.4 wt% V led to columnar-to-equiaxed transitions, and the average grain sizes were refined to 196 μm and 154 μm, respectively. The peritectic reaction Al_10_V particles were found near the grain center of α-Al. Rakhmonov et al. [23] studied the influence of Zr, V, and Ni additions on the solidification path and microstructure of Al–7Si–3Cu–0.3Mg alloy. They reported that the addition of vanadium yielded a grain refinement level similar to that of Zr-added alloy. However, the effect of vanadium on the solidification microstructure and mechanical properties of Al–Ni alloys has not been reported so far.

In this research, the effects of vanadium addition on the solidification microstructure and mechanical properties of hypoeutectic Al–4Ni alloy were studied via thermodynamic calculations, solidification curves, macro- and microstructural observations, and mechanical properties testing. The refining mechanism of the primary α-Al and eutectic structure by vanadium addition is discussed. The findings of this research are helpful to further understand the effects of transition element vanadium on the solidification microstructure and mechanical properties of hypoeutectic Al–4Ni alloy, and provide a basis for the development of novel Al–Ni heat-resistant aluminum alloys.

## 2. Experimental

**Figure 1:**
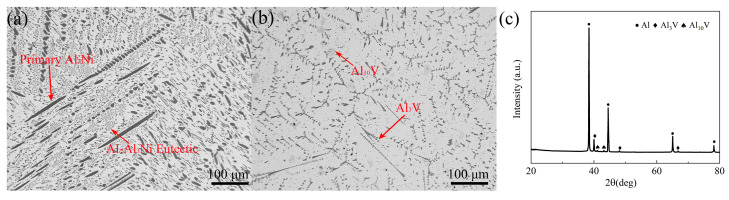
Microstructure and phase composition of the master alloys: (**a**) Al–10Ni, (**b**) Al–5V, (**c**) XRD of Al–5V.

Figure 1 shows the microstructure and phase composition of the master alloys. According to the phase diagram of Al–Ni [24], Al–10Ni alloy (Sichuan Lande Industry Co., Ltd., Chengdu, China) is composed of primary Al_3_Ni phases and Al–Al_3_Ni eutectic phases, whose microstructural morphologies are shown in Figure 1a. It can be seen from Figure 1b that the primary V-containing phases of Al–5V alloy (Sichuan Lande Industry Co., Ltd.) have two morphologies, which are dendrites and blocks. From the results of Figure 1c and ref. [25], it can be seen that the dendritic phase is Al_3_V and the block phase is Al_10_V.

**Figure 2:**
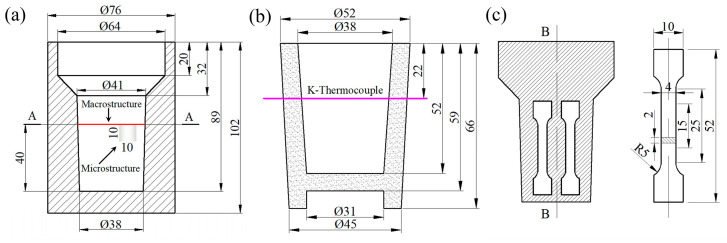
Schematic drawing of (**a**) permanent mold and sampling positions, (**b**) thermal analysis cup, (**c**) tensile sample (in mm).

Al–4 wt% Ni (abbr. Al–4Ni) alloys were prepared from commercial purity Al ingot (99.90 wt% Al) and Al–10 wt% Ni master alloy (0.04 wt% Si, 0.13 wt% Fe, 9.8 wt% Ni) using a 3-kilogram capacity ZnO-coated clay–graphite crucible in a resistance furnace (Yingshan Jianli electric furnace manufacturing Co., Ltd., Huanggang, China). After master alloy melting, the melt was heated to 800 ± 5 °C and was maintained for 1 h, during which it was stirred for 2 min every 10 min to ensure compositional homogeneity. Six additional vanadium levels in Al–4Ni–*x*V alloys (*x* = 0, 0.1, 0.2, 0.3, 0.4, 0.6 wt%) were melted by adding Al–5V (0.1 wt% Si, 0.14 wt% Fe, 0.03 wt% Ti, 0.01 wt% B, 5.01 wt% V) master alloy to the Al–4Ni melt. After the Al–5V master alloy was melted, the melt was held for 20 min, and then degassed using Ar for 10 min. The melt was subsequently poured into the permanent mold (cooling rate 7 °C/s) at 750 °C, as shown in Figure 2a. The permanent mold was preheated to 220 ± 5 °C.

The thermal analysis cup (cooling rate 2 °C/s) used in the experiment is shown in Figure 2b. The thermal analysis was conducted using a data acquisition instrument (NI–9213, National Instruments, Austin, TX, USA) with a cold-junction compensation accuracy of 0.8 °C, and a measurement sensitivity less than 0.02 °C. The measurement was carried out by recording the temperature range from 750 °C to 400 °C at a frequency of 75 Hz. DAQ–Express thermal analysis software was used to record the cooling curves.

The macrostructure samples were cut from the mid-transverse section at a height of 40 mm from the bottom (Figure 2a), and then prepared by grinding and polishing. The samples were etched using Keller’s reagent (190 mL H_2_O + 5 mL HNO_3_ + 3 mL HCl + 2 mL HF); a macro camera was used to capture the macrostructural images. The microstructure of the samples etched with 0.5% HF aqueous solution were observed with an optical microscope (DMM-580C, Caikon, Shanghai, China). In order to exclude the effect of cooling rate on microstructural observations, samples were taken from the same position of each ingot, which was 40 mm away from the bottom, and 7 mm away from the edge of the ingot (as shown in Figure 2a). The size of the primary α-Al grains was measured with a lineal intercept method using image analysis software (ImageJ^®^ 1.5.4), and an average value of ten visual fields was taken for each sample. Field emission scanning electron microscopy (FE–SEM, Nova Nano SEM 450, FEI, Eindhoven, The Netherlands) was used to observe the morphology of eutectic cells and the intermetallic compounds. The composition of intermetallic compounds and the distribution of alloying elements were analyzed using energy dispersive spectroscopy (EDS). In order to obtain the three-dimensional morphology of eutectic Al_3_Ni phases, each sample was deeply etched with a 10% HCl aqueous solution for 5–10 min to remove the α-Al around Al_3_Ni phases in eutectic cells, and observed using FSEM. Multiple fields of view were selected for each deep etching sample to take electron microscope photos; the diameters of at least 300 rod-like Al_3_Ni phases in the photos were measured via ImageJ, and the average diameter of the Al_3_Ni phases was calculated.

Phase analysis was carried out with X-ray diffraction (XRD–7000S X–ray diffractometer, Shimadzu, Kyoto, Japan) using Cu *K*_a_ radiation. The diffraction patterns were obtained at a voltage of 40 kV, in the 2*θ* range of 20–80°, with a scanning speed of 2°/min.

The tensile test was performed on a universal testing machine (AG-IC/100kN, Shimadzu, Japan Shimadzu AG-100kN, Kyoto, Japan) with a loading speed of 1.0 mm/min. The sampling position and size of the tensile sample are shown in Figure 2c. The tensile strength and elongation were obtained from the average of three samples. The tensile fracture morphology was observed using FE-SEM.

The microhardness measure of the alloys was conducted with a load of 4.9035 N for 30 s on a Vickers hardness tester (430 SVD, Wilson, NC, USA). From each sample, 10 points were randomly selected for measurement, from which the average value was calculated.

## 3. Results and Discussion

### 3.1. Thermodynamic Analyses of the Al–4Ni–V System

**Figure 3:**
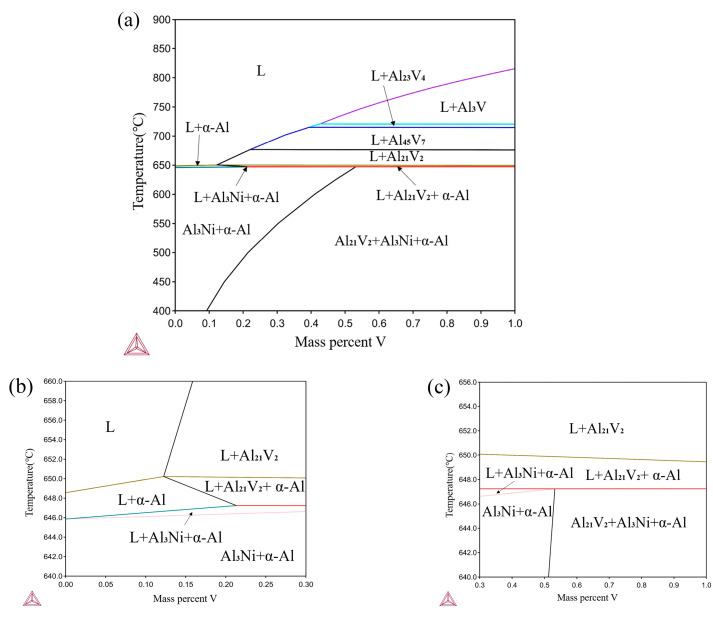
Al–4Ni–(0–1)V phase diagram simulated by Thermo-Calc: (**a**) Al–4Ni–(0–1)V, (**b**) 0–0.3 wt% V local diagram, (**c**) 0.3–1 wt% V local diagram.

Although the Ni–Al–V ternary phase diagram has been studied for Ni–Al intermetallic compounds since the 1980s [26], the pseudobinary phase diagram is only the Ni_3_Al–Ni_3_V part [27]. There is a lack of phase diagram studies for Al–Ni–V alloys with high Al content. Therefore, the phase diagram of the Al–4Ni–(0–1.0)V ternary system was determined using the commercial thermodynamics software (Thermo-Calc^®^ 2022b, Database TCAL 8), as shown in Figure 3. As can be seen from Figure 3a, when vanadium additions are 0.42–1.0 wt%, peritectic reactions such as those shown in Equations (1)–(4) occur successively. The phase compositions of Al–4Ni–(0–1)V alloy at room temperature are α-Al, Al_3_Ni, and Al_21_V_2_ (also designated as Al_10_V [28]). Compared with the Al–V binary phase diagram [28], the peritectic point composition and peritectic temperature (Equation (4)) in Al–4Ni–(0–1)V alloys decrease from 0.17 *wt%* *V* and 660 °C for Al–V alloy to 0.12 *wt%* *V* and 650 °C, respectively.

(1) $$L_{0.42wt\%V}+{\mathrm{Al}}_{3}V\to {\mathrm{Al}}_{23}V_{4}\;(724\;{}^{\circ}C)$$

(2) $$L_{0.39wt\%V}+{\mathrm{Al}}_{23}V_{4}\to {\mathrm{Al}}_{45}V_{7}\;(717\;{}^{\circ}C)$$

(3) $$L_{0.22wt\%V}+{\mathrm{Al}}_{45}V_{7}\to {\mathrm{Al}}_{21}V_{2}\;(678\;{}^{\circ}C)$$

(4) $$L_{0.12wt\%V}+{\mathrm{Al}}_{21}V_{2}\to \alpha \text{-Al}\;(650\;{}^{\circ}C)$$

It can be seen from Figure 3b,c that the eutectic solidification of Al–4Ni–(0–1)V alloy occurs within a certain temperature range. The liquidus temperature of the Al–Al_3_Ni eutectic alloy increases with the vanadium additions (0–0.21 wt%), and there is no significant change when the vanadium amount exceeds 0.21 wt%. The final temperature of the eutectic solidification increases with increased vanadium additions. When the vanadium additions are higher than 0.5 wt%, the difference between the initial temperature and the final temperature of the eutectic solidification is very small.

### 3.2. Solidification Process

**Figure 4:**
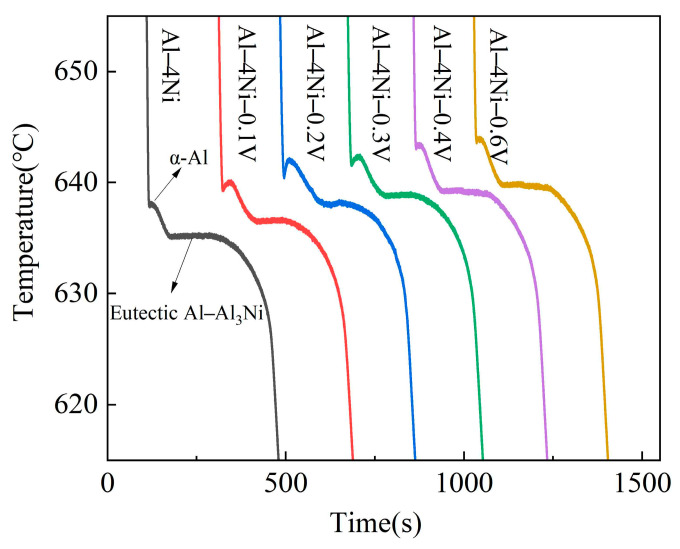
Cooling curves of Al–4Ni–xV alloys.

**Figure 5:**
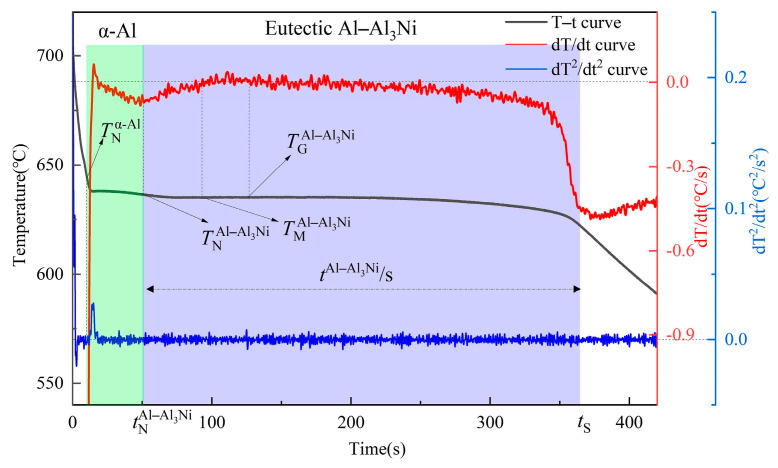
Cooling curves of Al–4Ni alloy and differential transformations.

To investigate the effect of vanadium addition on the solidification process of Al–4Ni alloys, temperature–time curves were collected during the solidification process of Al–4Ni alloys with different vanadium additions, as shown in Figure 4. Figure 5 shows a typical cooling curve of Al–4Ni alloy. The first derivative and second derivative of the cooling curves were calculated, and the solidification characteristic parameters were determined. $T_{N}^{\alpha \text{-Al}}$ and $T_{N}^{\mathrm{Al}–{\mathrm{Al}}_{3}\mathrm{Ni}}$ are the nucleation temperatures of the primary α-Al and Al–Al_3_Ni eutectic alloys, respectively. $T_{G}^{\mathrm{Al}–{\mathrm{Al}}_{3}\mathrm{Ni}}$ and $T_{M}^{\mathrm{Al}–{\mathrm{Al}}_{3}\mathrm{Ni}}$ are the growth temperature and minimum temperature of the Al–Al_3_Ni eutectic solidification, respectively, while $t^{\mathrm{Al}–{\mathrm{Al}}_{3}\mathrm{Ni}}$ is the time for Al–Al_3_Ni eutectic solidification.

Table 1 shows the characteristic parameters of primary α-Al and eutectic solidifications of Al–4Ni alloys with different vanadium additions. For the solidifications of primary α-Al, it can be seen that with the vanadium addition, the $T_{N}^{\alpha \text{-Al}}$ increased gradually. When vanadium added was 0.6 wt%, the $T_{N}^{\alpha \text{-Al}}$ increased from 642.5 °C for the Al–4Ni alloy to 646.5 °C.

For the eutectic solidification, the data show that the $T_{N}^{\mathrm{Al}–{\mathrm{Al}}_{3}\mathrm{Ni}}$ increased with increased vanadium addition. When the vanadium addition was 0.2 wt%, the $T_{N}^{\mathrm{Al}–{\mathrm{Al}}_{3}\mathrm{Ni}}$ increased from 636.2 °C for the Al–4Ni alloy to 640.5 °C. As the vanadium addition continued to increase, the $T_{N}^{\mathrm{Al}–{\mathrm{Al}}_{3}\mathrm{Ni}}$ did not change significantly. When vanadium additions were less than 0.3 wt%, the recalescence temperature (${∆T}_{R}^{\mathrm{Al}–{\mathrm{Al}}_{3}Ni}=T_{G}^{\mathrm{Al}–{\mathrm{Al}}_{3}\mathrm{Ni}}-T_{M}^{\mathrm{Al}–{\mathrm{Al}}_{3}\mathrm{Ni}}$) increased, and the $t^{\mathrm{Al}–{\mathrm{Al}}_{3}\mathrm{Ni}}$ decreased. When the vanadium addition was 0.2 wt%, the ${∆T}_{R}^{\mathrm{Al}–{\mathrm{Al}}_{3}Ni}$ increased from 0.2 °C for the Al–4Ni alloy to 0.7 °C, and the $t^{\mathrm{Al}–{\mathrm{Al}}_{3}\mathrm{Ni}}$ decreased from 310 s to 282 s. However, as the vanadium addition continued to increase, the ${∆T}_{R}^{\mathrm{Al}–{\mathrm{Al}}_{3}Ni}$ began to decrease, and the $t^{\mathrm{Al}–{\mathrm{Al}}_{3}\mathrm{Ni}}$ increased. When the vanadium addition was 0.6 wt%, the ${∆T}_{R}^{\mathrm{Al}–{\mathrm{Al}}_{3}Ni}$ decreased to 0.3 °C, and the $t^{\mathrm{Al}–{\mathrm{Al}}_{3}\mathrm{Ni}}$ increased to 302 s. This is consistent with the effect of a third element on the eutectic solidification reported in ref. [29]; when the addition of a third element is low, the growth rate increases in a certain supercooling range compared with the solidification of the binary eutectic alloy, while the growth rate decreases in all supercooling ranges when the addition of a third element is high enough.

### 3.3. XRD Phase Analysis

**Figure 6:**
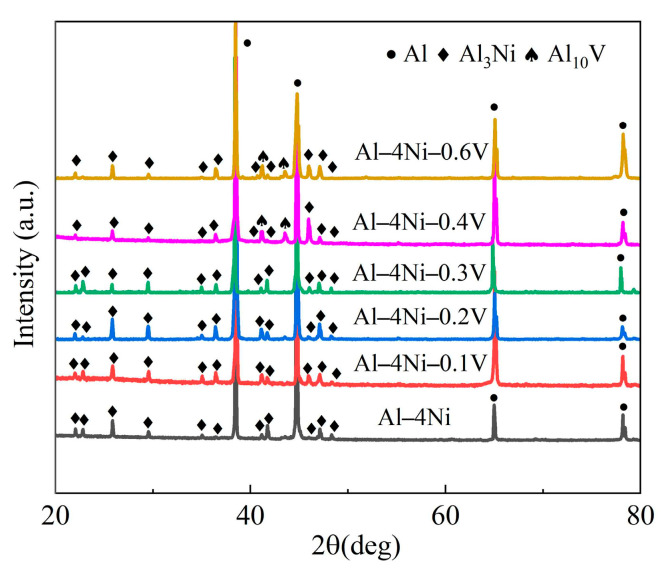
XRD patterns of Al–4Ni–*x*V alloys.

Figure 6 shows the XRD patterns of the Al–4Ni alloys with different vanadium additions. The results show that the diffraction peaks in the Al–4Ni alloy are the α-Al phase and the Al_3_Ni phase. When the vanadium additions are in the range of 0.1–0.3 wt%, the phase composition phases are still the α-Al phase and the Al_3_Ni phase. New diffraction peaks appear in the Al–4Ni alloys with 0.4 wt% V and 0.6 wt% V, which were confirmed to be Al_10_V intermetallic compounds by the powder diffraction file (PDF) database. This is consistent with the phase composition in the phase diagram in Figure 3.

### 3.4. Microstructural Evolution

**Figure 7:**
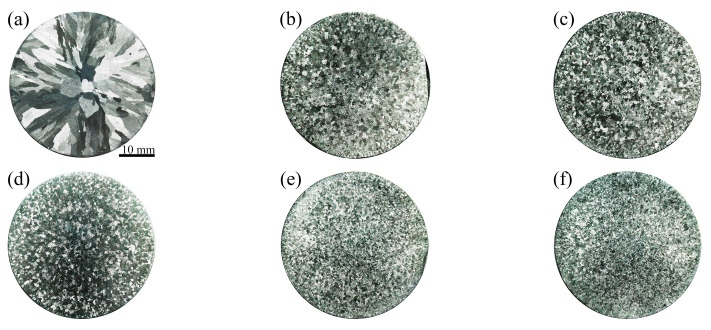
Macrostructures of Al–4Ni alloys with different vanadium additions: (**a**) 0 wt%, (**b**) 0.1 wt%, (**c**) 0.2 wt%, (**d**) 0.3 wt%, (**e**) 0.4 wt%, (**f**) 0.6 wt%.

Figure 7 shows macrostructures of cross sections of the Al–4Ni–*x*V alloy samples. It can be seen from Figure 7a that Al–4Ni alloy is composed of coarse columnar grains, and the grains show radial and slender morphologies, which are consistent with the radial solidification direction from the mold wall to the center. With the addition of 0.1 wt% V (Figure 7b), the macrostructure of the Al–4Ni–0.1V alloy changed from columnar to equiaxed grains. The grain sizes of the Al–4Ni–*x*V alloys decreased with increased vanadium addition, when the vanadium additions were 0.4 wt% and 0.6 wt%; the grain sizes were difficult to clearly distinguish by the macrostructure.

**Figure 8:**
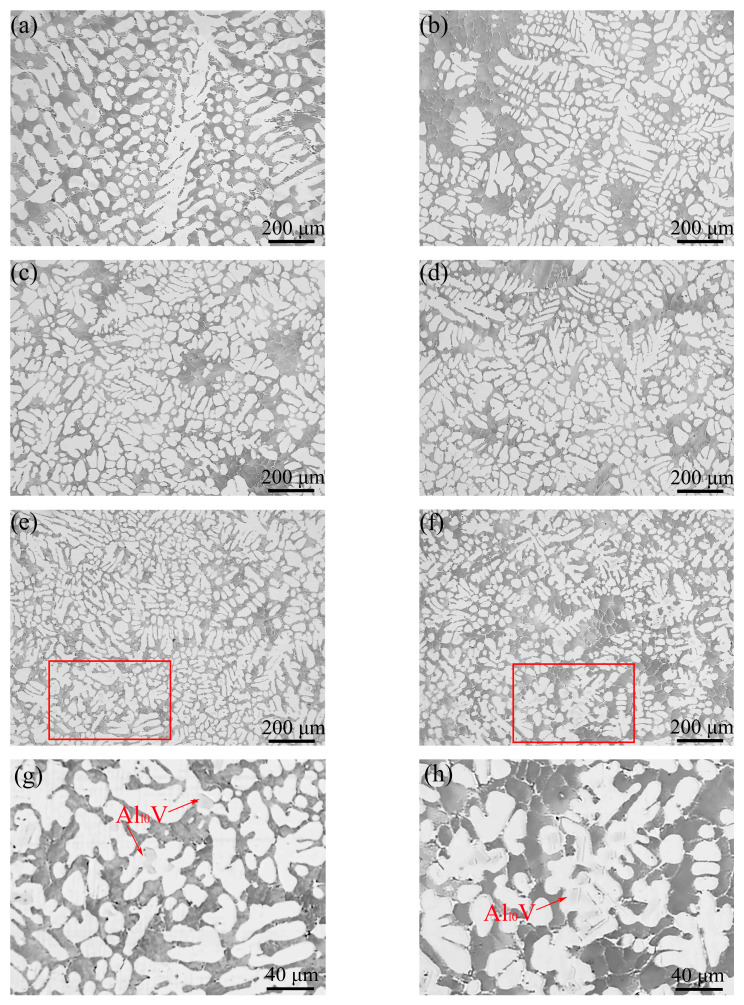
Morphologies of the primary α-Al in Al–4Ni–*x*V alloys. (**a**) 0 wt%, (**b**) 0.1 wt%, (**c**) 0.2 wt%, (**d**) 0.3 wt%, (**e**) 0.4 wt%, (**f**) 0.6 wt%, (**g**) rectangular area in (**e**), (**h**) rectangular area in (**f**).

The morphologies of the primary α-Al in the Al–4Ni–*x*V alloys are shown in Figure 8. As can be seen in Figure 8a, the primary α-Al of the Al–4Ni alloy is coarse dendrite. A transition from columnar to equiaxed growth took place when adding vanadium to the Al–4Ni alloys, and the sizes were significantly reduced, although there were still a few primary α-Al dendrites. It can be seen from Figure 8e–h that granular intermetallic compounds appear inside the primary α-Al grains when the vanadium additions are 0.4 wt% and 0.6 wt%. From the XRD phase analysis in Figure 6, the compounds should be Al_10_V. From Figure 6 and Figure 8, it can be determined that no Al_10_V phases exist in the Al–4Ni alloys with vanadium additions less than 0.4 wt%. This is because non-equilibrium solidification increases the solid solubility of vanadium in α-Al, and a small amount of vanadium decomposed by the peritectoid reaction of Al_10_V was solidly dissolved in α-Al.

**Figure 9:**
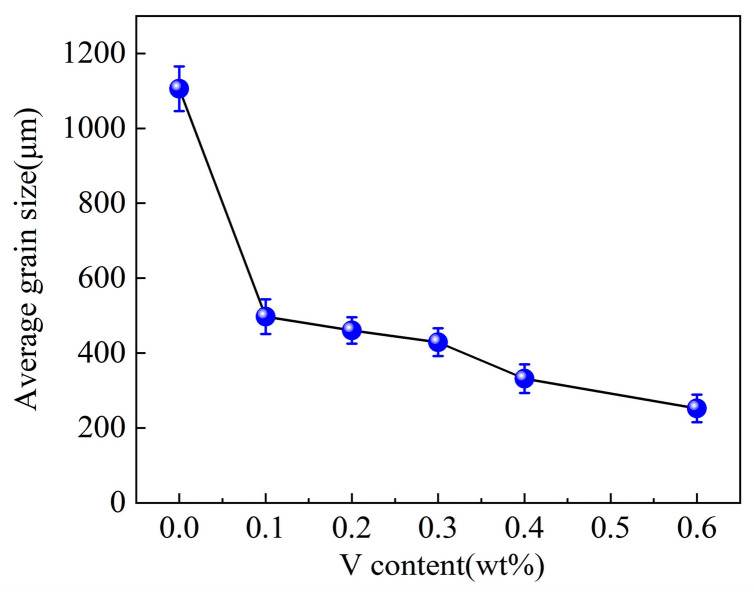
Average grain sizes of Al–4Ni alloys with different vanadium additions.

Figure 9 shows the average grain sizes of the Al–4Ni alloys with different vanadium additions. The results show that the average grain size of primary α-Al in the sample with 0.1 wt% V decreased from 1105 μm of Al–4Ni alloy to 497 μm. With increasing vanadium addition, the average grain sizes of α-Al decreased gradually, and the grain size of the alloy with 0.6 wt% V was refined to 252 μm. This indicates that vanadium addition causes equiaxation of the primary α-Al grains of Al–4Ni alloys, effectively refining the grains.

As reported in ref. [30], supercooling caused by solute elements makes an important contribution to effective grain refinement. When the vanadium additions were less than 0.3 wt%, the constitutional supercooling due to the addition of vanadium at the crystallization front provided the driving force for the nucleation of primary α-Al. Thus, as the nucleation of primary α-Al was advanced, the $T_{N}^{\alpha \text{-Al}}$ increased. Meanwhile, as reported in ref. [31], the α-Al average grain size is inversely proportional to the growth restriction factor (*Q*) based on constitutional supercooling theory. According to the Al–V binary phase diagram [32], the liquidus slope *m*_L_ is 9.71 K/wt%, the equilibrium partition coefficient *k* is 3.29, and the calculated *m*_L_ (*k* − 1) is 22.24 K/wt%. The growth-limiting factor *Q* values of the alloys with 0.1 wt% V and 0.2 wt% V were 2.22 K and 4.45K, respectively. The growth-restricting effect of vanadium effectively restrained the growth of primary α-Al.

On the other hand, as shown in Figure 3, when the vanadium additions exceeded 0.21 wt%, the primary Al_10_V compounds were precipitated from the liquid. Al_10_V has the same face-centered cubic as α-Al crystal, with a lattice constant of 1.4492 nm, an Fd3m space group, and contains 160 Al atoms and 16 V atoms in each unit cell [33]. There are the matching relationships between Al_10_V particles and Al crystal in three orientation relationships: [22] (1) [10-1]Al//[11-2]${\mathrm{Al}}_{10}V$, (020)Al//(444)${\mathrm{Al}}_{10}V$; (2) [01-1]Al//[12-3]${\mathrm{Al}}_{10}V$, (111)Al//(444)${\mathrm{Al}}_{10}V$; and (3) [10-1]Al//[10-1]${\mathrm{Al}}_{10}V$, (111)Al//(444)${\mathrm{Al}}_{10}V$. Among them, the relationship between (020)Al and (444)${\mathrm{Al}}_{10}V$ has the smallest interatomic spacing misfit, (*f*_r_ = 3.2%) and interplanar spacing mismatch (*f*_d_ = 3.15%). Therefore, when the vanadium additions exceeded 0.2 wt%, the number of Al_10_V particles increased, and the heterogeneous nucleation rate increased significantly; the grains were refined gradually.

**Figure 10:**
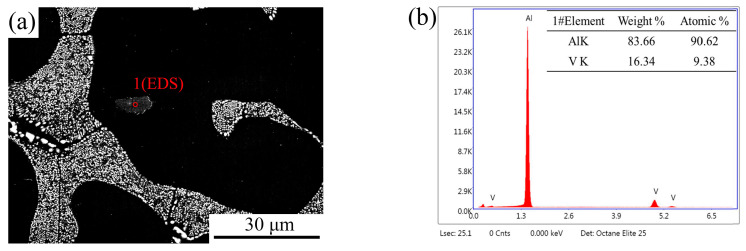
Al_10_V particle in Al–4Ni–0.6V alloy: (**a**) particle morphology; (**b**) EDS analysis of point 1.

Figure 10 shows the morphology and EDS composition of Al_10_V particles in the Al–4Ni–0.6V alloy. From Figure 3 and Figure 10, it can be found that the particles should be residual Al_10_V from the hyper-peritectic reaction. This indicates that the heterogeneous nucleation of primary α-Al was based on Al_10_V particles.

Based on the above results, it can be inferred that the constitutional supercooling formed at low vanadium additions and the heterogeneous nucleation of Al_10_V particles formed at high vanadium additions in Al liquid can both play an important role in refining the primary α-Al grains.

**Figure 11:**
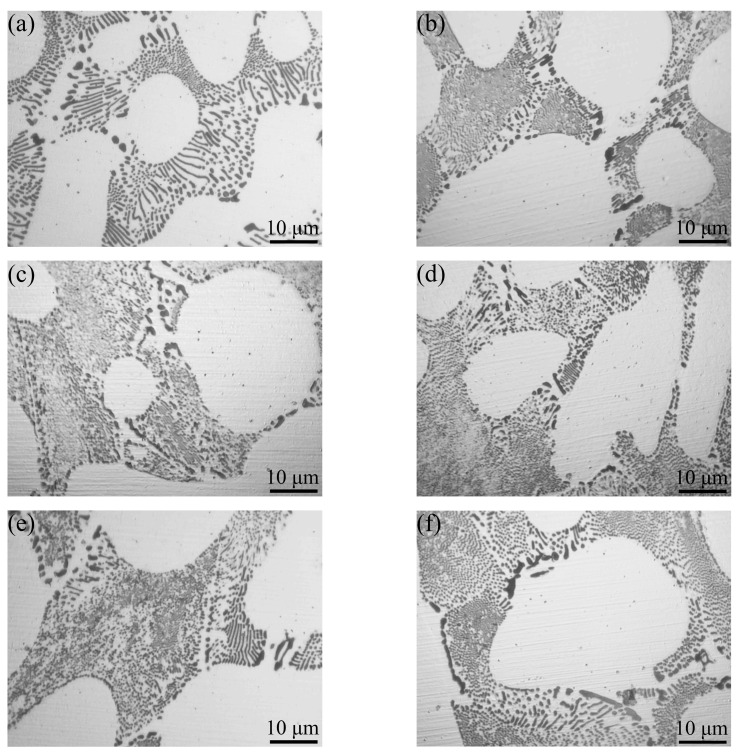
Eutectic morphologies of Al–4Ni alloys with different vanadium additions: (**a**) 0 wt%, (**b**) 0.1 wt%, (**c**) 0.2 wt%, (**d**) 0.3 wt%, (**e**) 0.4 wt%, (**f**) 0.6 wt%.

Figure 11 shows the eutectic structure of the Al–4Ni–*x*V alloys. As shown in Figure 11a, the eutectic cells of the Al–4Ni alloy are composed of central rod-like eutectic and boundary lamellar eutectic. There are coarse granular eutectic Al_3_Ni phases at the boundary of eutectic cells, which are caused by the segregation of alloy element Ni during the solidification process. In the Al–4Ni alloys with 0.1 wt% and 0.2 wt% V (Figure 11b,c), the eutectic cells are mainly rod-like eutectic, and the lamellar eutectic and coarse granular eutectic Al_3_Ni phases in the eutectic boundaries are significantly reduced, among which the size of eutectic Al_3_Ni phase in the sample with 0.2 wt% is the smallest. When the vanadium additions exceeded 0.2 wt% (Figure 11d–f), the size of Al_3_Ni phases in the eutectic cells began to increase, and the number of lamellar eutectic and granular Al_3_Ni phases in the eutectic boundaries increased, but the sizes of these eutectic Al_3_Ni phases were smaller than that of the Al–4Ni alloy. These results indicate that an appropriate vanadium addition can effectively refine the eutectic structure of Al–4Ni alloys.

**Figure 12:**
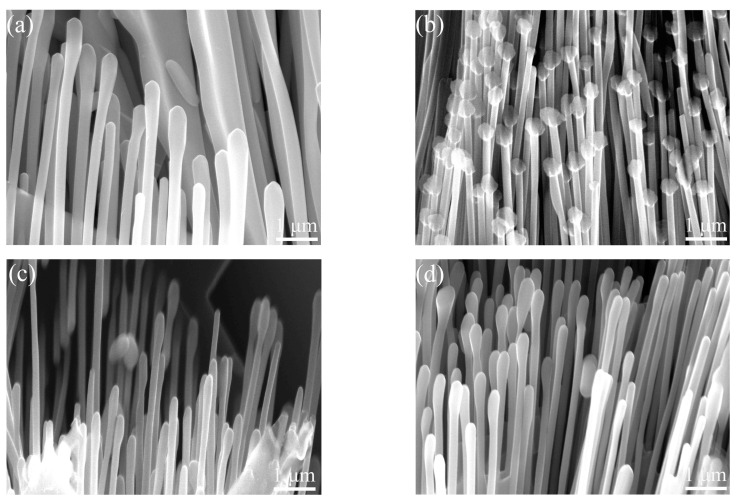
Three-dimensional morphologies of Al_3_Ni phases with different vanadium additions: (**a**) 0 wt%; (**b**) 0.2 wt%; (**c**) 0.4 wt%; (**d**) 0.6 wt%.

Figure 12 shows three-dimensional morphologies of the Al_3_Ni phase with different vanadium additions. It can be seen from Figure 12a that the eutectic Al_3_Ni phases of Al–4Ni alloy are composed of rod-like and lamellar morphologies, and the lamellar spacing is significantly larger than the rod-like spacing. This structure of Al–4Ni eutectic cells is consistent with the results reported in ref. [34]. Kakitani et al. [35] referred to this type of eutectic morphology with different phase spacings as a bimodal structure. However, the morphology of the Al_3_Ni phases in the samples with vanadium addition (Figure 12b–d) is rod-like, with no obvious lamellar Al_3_Ni phase, indicating that the growth mode and morphology of the eutectic Al_3_Ni phases were modified by the vanadium addition.

Comparing Figure 12b–d, it can be found that the fibrosis of Al_3_Ni phases in the Al–4Ni alloy with 0.2 wt% V are more obvious than that of the alloy with 0.4 wt% V and 0.6 wt% V. The results of quantitative statistics show that the average diameter of Al_3_Ni phases in the Al–4Ni alloy is 0.26 μm, while the average diameter of Al–4Ni alloy with 0.2 wt% V is 0.14 μm, which are reduced by 46%. However, when the vanadium additions increased to 0.4 wt% and 0.6 wt%, the average diameters of the Al_3_Ni phases increased to 0.16 μm and 0.20 μm, respectively. This is because the $t^{{\mathrm{Al}–\mathrm{Al}}_{3}\mathrm{Ni}}$ increases (as shown in Table 1) and the eutectic phases have enough time to grow when the vanadium additions exceed 0.2 wt%. In addition, the decrease in ${∆T}_{R}^{{\mathrm{Al}–\mathrm{Al}}_{3}\mathrm{Ni}}$ indicates that the latent heat of crystallization decreases during the eutectic solidification, which reflects the decrease in the nucleation rate of eutectic Al_3_Ni phases. This is similar to the results reported by Gan et al. [15]. Their study showed that trace Co can significantly refine the eutectic Al_3_Ni phases in the Al–2.5Ni alloy, and the coarsening of the eutectic Al_3_Ni phases begins when the Co content exceeds 0.1 at%.

**Figure 13:**
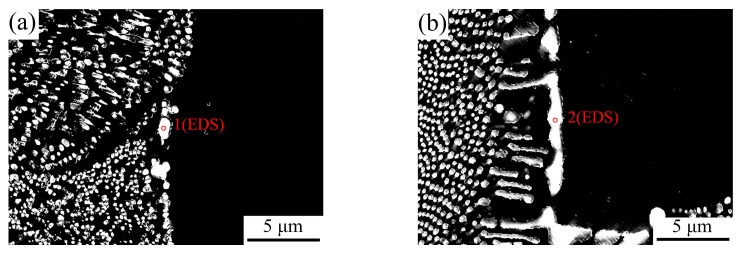
The Al_3_Ni phases at the boundary of eutectic cells in Al–4Ni–0.2V and Al–4Ni–0.6V alloy: (**a**) Al–4Ni–0.2V, (**b**) Al–4Ni–0.6V.

Figure 13 shows the Al_3_Ni phases at the boundary of eutectic cells in the Al–4Ni–0.2V and Al–4Ni–0.6V alloys, and the EDS composition of Al_3_Ni phases is shown in Table 2. As can be seen from Figure 13a, the eutectic boundary of Al–4Ni–0.2V alloy contains only a small amount of coarse particle Al_3_Ni phases, while the eutectic boundary of Al–4Ni–0.6V alloy forms coarse lamellar Al_3_Ni phases (Figure 13b), indicating that a high amount of vanadium addition segregates at the eutectic cell boundaries and coarsens the eutectic Al_3_Ni phases. The composition in Table 2 shows that only a small amount of vanadium dissolved in the Al_3_Ni phases, while more vanadium dissolved in the Al_3_Ni phase at the eutectic boundary of the Al–4Ni–0.6V alloy. On the other hand, the Fe impurity segregated at the boundary of eutectic cell was more dissolved in the Al_3_Ni phases of the Al–4Ni–0.6V alloy, and no iron-rich phases were found in the microstructure.

Based on the morphologies of the eutectic phases, the characteristic parameters of the solidification curves, and the distribution of elements in the eutectic cell, the following mechanism for the influence of vanadium on eutectic solidification is proposed. During the solidification process of Al–4Ni alloy with vanadium addition, alloying elements V and Ni segregate at the interfaces between the α-Al and liquid with the growth of the primary α-Al phase. As a result of the electronegativity difference in the Al–V being greater than that in the Al–Ni (electronegativities of Al, Ni, and V are 2.52, 2.32, and 2.08, respectively [36]), vanadium segregates at the interface between the primary α-Al and liquid formed during constitutional supercooling. This provides a driving force for the nucleation of the eutectic leading phase—Al_3_Ni—which increases the $T_{N}^{\mathrm{Al}–{\mathrm{Al}}_{3}\mathrm{Ni}}$ and leads to fibrosis of the Al_3_Ni phase. When the vanadium additions are less than 0.2 wt%, the segregation of alloying elements at the eutectic crystallization front is less, due to the shortening of the $t^{\mathrm{Al}–{\mathrm{Al}}_{3}\mathrm{Ni}}$. Therefore, the distribution of alloying elements in eutectic cells is relatively uniform, and there are no obvious coarse lamellar Al_3_Ni phases at the boundary of eutectic cells, as shown in Figure 13a. When the vanadium additions exceed 0.2 wt%, due to the increase in the $t^{\mathrm{Al}–{\mathrm{Al}}_{3}\mathrm{Ni}}$, the content of alloying elements in the final solidified eutectic liquid (as shown in Table 2) increase. At the same time, the precipitation of primary Al_10_V in the liquid phase increases, and the vanadium content at the solid–liquid interface in the eutectic decrease further. As a result, the morphologies of the boundary Al_3_Ni phases change from particle to lamellar (as shown in Figure 13b), and the eutectic structure coarsens.

Therefore, 0.2 wt% V can increase nucleation and the recalescence temperature of eutectic mixture, shorten eutectic solidification time, significantly refine eutectic Al_3_Ni phases, while excessive vanadium leads to coarsening of the eutectic Al_3_Ni phases.

### 3.5. Mechanical Properties

**Figure 14:**
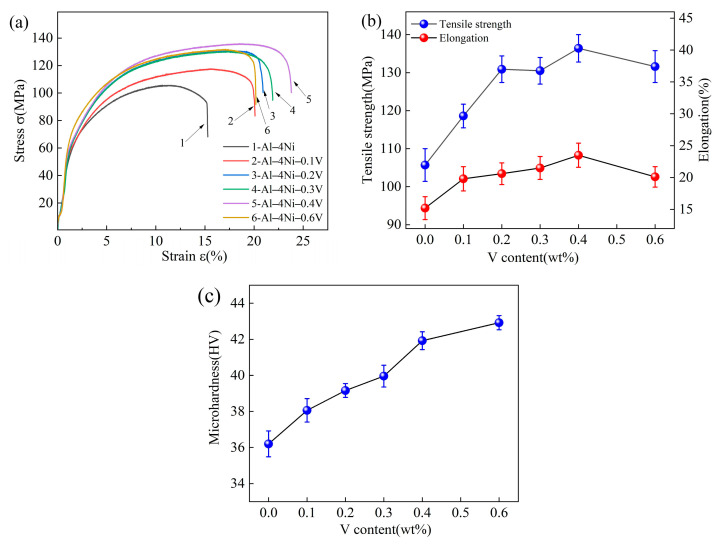
Mechanical properties of Al–4Ni–*x*V: (**a**) stress–strain curves, (**b**) tensile strength and elongation, (**c**) microhardness.

Figure 14 shows the stress–strain curves and tensile properties of the Al–4Ni–*x*V alloys. As can be seen from Figure 14a, the tensile strength and elongation of Al–4Ni alloy improve with the addition of vanadium. As can be seen from Figure 14b, the tensile strength and elongation of the Al–4Ni–0.4V alloy increased from 105.7 MPa and 15.2% of the Al–4Ni alloy to 136.4 MPa and 23.5%, which are 29.1% and 54.6% higher than those of the Al–4Ni alloy, respectively. When the vanadium addition was 0.6 wt%, the tensile strength and elongation began to decrease. Figure 14c shows the microhardness of the Al–4Ni–*x*V alloy. The results show that with increased vanadium addition, the microhardness of the Al–4Ni alloy increased from 36 HV to 43 HV. Grain refinement and primary Al_10_V particles are the main reasons for this increase in the microhardness.

The above results show that the addition of vanadium can significantly improve the mechanical properties of Al–4Ni alloys. There are two reasons; firstly, vanadium can significantly refine the primary α-Al phase of hypoeutectic Al–4Ni alloy. Grain refinement can significantly improve the strength of the alloy [37]. On the other hand, grain refinement is beneficial to restrain crack formation during plastic deformation, which also improves the elongation [38,39].

Secondly, an appropriate amount of vanadium can refine the eutectic Al_3_Ni phases; the thinning of rod-like eutectic structures result in the decrease in the eutectic spacing. The smaller eutectic spacing is also conducive to improvements in the mechanical properties of Al–4Ni alloy [40]. According to Figure 8 and Figure 11, we found that the Al–4Ni–0.2V alloy had a fine eutectic structure, but the average grain size of primary α-Al was thick, which limits its mechanical properties. Compared with the Al–4Ni–0.2V alloy, the Al–4Ni–0.4V alloy contained a small amount of lamellar eutectic mixture at the boundary of the eutectic cells, but the average size of rod-like Al_3_Ni phases in the eutectic cells was similar to that of the Al–4Ni–0.2V alloy, and the degree of refinement of primary α-Al was higher. Therefore, adding 0.4 wt% V to Al–4Ni alloy can result in better mechanical properties.

However, when vanadium addition increased to 0.6 wt%, more Al_10_V phases existed in the matrix of the Al–4Ni–0.6V alloy than those in the Al–4Ni–0.4V alloy. The Al–4Ni–0.6V alloy contains the hard phases, such as primary Al_10_V and coarse Al_3_Ni at the eutectic boundaries. The plastic deformation of these hard phases was inconsistent with that of the α-Al matrix. Therefore, during the plastic deformation process, microcracks formed between these hard phases and the α-Al matrix. The propagation, connection, and link-up of microcracks led to the formation of local cleavage fracture, resulting in a decrease in both the strength and plasticity of the Al–4Ni–0.6V alloy.

**Figure 15:**
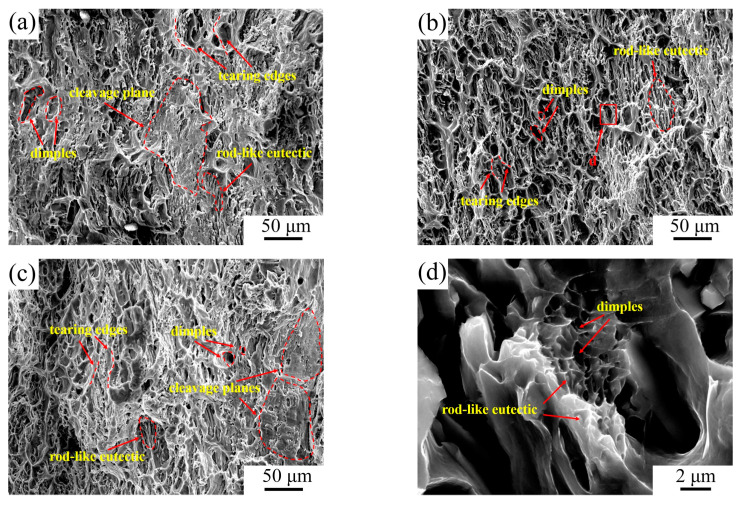
Fracture morphologies of Al–4Ni–*x*V alloys: (**a**) Al–4Ni, (**b**) Al–4Ni–0.4V, (**c**) Al–4Ni–0.6V, (**d**) inside of Al–4Ni–0.4V dimples.

Figure 15 shows the fracture morphologies of Al–4Ni alloys with different vanadium additions. As shown in Figure 15a, the fracture morphology of Al–4Ni alloy is composed of tear edges, a few dimples and cleavage planes, and exhibits a mixed ductile–brittle fracture, which is caused by coarse α-Al dendrites and eutectic Al_3_Ni phases. The Al–4Ni–0.4V alloy (Figure 15b) shows a typical ductile fracture, with obvious plastic deformation of the grain on the fracture, which is composed of a large number of tearing edges and dimples with different sizes. The enlarged fracture observed in the region of the eutectic cells (Figure 15d) shows that there are many small dimples that are centered on Al_3_Ni phases in the region of the eutectic cells, indicating that the α-Al in the eutectic cells also undergoes plastic deformation. It can be seen that refining the primary α-Al and eutectic Al_3_Ni phases can significantly improve the plasticity of Al–4Ni alloy. As can be seen from Figure 15c, the fracture surface of Al–4Ni–0.6V is composed of dimples, tearing edges, and cleavage planes, and exhibits a mixed ductile–brittle fracture. Cleavage planes form due to local brittle fracture of Al_10_V and coarse Al_3_Ni phases.

## 4. Conclusions

This research examined the effects of vanadium addition on the solidification process, microstructure evolution, and mechanical properties of Al–4Ni alloy. The main conclusions are as follows:

(1) With increased vanadium addition (0–0.6 wt%), the $T_{N}^{\alpha \text{-Al}}$ increased. When the vanadium addition was 0.6 wt%, the $T_{N}^{\alpha \text{-Al}}$ increased from 642.5 °C for the Al–4Ni alloy to 646.5 °C.
(2) When the vanadium addition was 0.2 wt%, the $T_{N}^{\mathrm{Al}–{\mathrm{Al}}_{3}\mathrm{Ni}}$ and ${∆T}_{R}^{\mathrm{Al}–{\mathrm{Al}}_{3}\mathrm{Ni}}$ increased from 636.2 °C and 0.2 °C for the Al–4Ni alloy to 640.5 °C and 0.7 °C, respectively. The $t^{\mathrm{Al}–{\mathrm{Al}}_{3}\mathrm{Ni}}$ decreased from 310 s to 282 s. As the vanadium addition continued to increase, the $T_{N}^{\mathrm{Al}–{\mathrm{Al}}_{3}\mathrm{Ni}}$ did not change significantly, and the $t^{\mathrm{Al}–{\mathrm{Al}}_{3}\mathrm{Ni}}$ began to increase.
(3) The columnar-to-equiaxed transition of primary α-Al took place when adding vanadium to the Al–4Ni alloy. The average grain size of the primary α-Al reduced from 1105 μm to 252 μm when the vanadium addition was increased from 0 to 0.6 wt%.
(4) When the vanadium addition was less than 0.2 wt%, the eutectic structure was refined. The average diameter of the eutectic Al_3_Ni phases in the Al–4Ni–0.2V alloy was 0.14 μm, which was 46% lower than 0.26 μm for the Al–4Ni alloy. As the vanadium additions became higher than 0.2 wt%, the eutectic Al_3_Ni phases began to coarsen again.
(5) The mechanical properties of Al–4Ni alloys can be improved with the addition of vanadium. The Al–4Ni–0.4V alloy obtained the highest tensile strength and elongation of 136.4 MPa and 23.5%, which were 29.1% and 54.6% higher than that of the Al–4Ni alloy, respectively. When the vanadium addition was 0.6 wt%, the tensile strength and elongation decreased. The microhardness of the Al–4Ni alloys increased gradually with increased additions of vanadium.
(6) The fracture of the Al–4Ni–0.4V alloy was composed of dimples and tear edges, demonstrating ductile fracture. Meanwhile, the fracture of the Al–4Ni–0.6V alloy was composed of dimples, tear edges, and cleavage planes, showing mixed ductile–brittle fracture. The cleavage planes were caused by the primary Al_10_V and coarse Al_3_Ni phases at the boundary of the eutectic cells.

## Figures and Tables

**Table 1:** Characteristic parameters of primary α-Al and Al–Al_3_Ni eutectic solidification.

V (wt%)	$T_{N}^{\alpha \text{-Al}}$ (°C)	$T_{N}^{\;\mathrm{Al}–{\mathrm{Al}}_{3}\mathrm{Ni}}$ (°C)	$T_{G}^{\;\mathrm{Al}–{\mathrm{Al}}_{3}\mathrm{Ni}}$ (°C)	$T_{M}^{\;\mathrm{Al}–{\mathrm{Al}}_{3}\mathrm{Ni}}$ (°C)	${∆T}_{R}^{\;\mathrm{Al}–{\mathrm{Al}}_{3}\mathrm{Ni}}$ (°C)	$t^{\;\mathrm{Al}–{\mathrm{Al}}_{3}\mathrm{Ni}}$ (s)
0	642.5	636.2	635.1	634.9	0.2	310
0.1	643.4	638.3	636.8	636.3	0.5	293
0.2	644.1	640.5	638.5	637.8	0.7	282
0.3	645.2	640.6	639.1	638.7	0.4	293
0.4	646.4	640.8	639.4	639.1	0.3	298
0.6	646.5	641	639.9	639.6	0.3	302

**Table 2:** EDS analysis of point 1 and point 2 in Figure 13.

Point	Al (at%)	Ni (at%)	V (at%)	Fe (at%)
1	79.14	20.36	0.15	0.35
2	77.29	21.53	0.45	0.73

## Data Availability

Data are contained within the article.
